# Supplementary material for: Halogenated Dihydropyrrol-2-One Molecules Inhibit Pyocyanin Biosynthesis by Blocking the Pseudomonas Quinolone Signaling System
Source: Molecules. 2022 Feb 9;27(4):1169. doi: 10.3390/molecules27041169 (PMC8875348; doi:10.3390/molecules27041169)
Supplement: Supplementary file 1 [file molecules-27-01169-s001.zip › molecules-1548863-supplementary.pdf]

Table S1. summarizes the average pyocyanin production range by *P. aeruginosa* when grown in presence of DHPs at concentrations ranging from 12.5 to 50 μg/ml.

***P. aeruginosa* strains**	**Pyocyanin biosynthesis in presence of DHP compounds (12.5 -50 μg/ml)**			
	**W1-Br**	**W1-Cl**	**W1-F**	**W1**
PAO1	61-25 %	83-40 %	68-21 %	90-59 %
MH602	41-23 %	52-31%	69-18%	47-25%
ATCC 25619	22-5 %	33-7 %	15-6 %	48-9 %
DFU-53 leg ulcer	94-55 %	77-51 %	88-57%	75-52%
364707 wound	83-38 %	64-24 %	49-16 %	71-39 %
**Overall performance**	60-29 %	62-31 %	58-24 %	66-37 %
